# Thickness-Dependent Permeation Properties of Quenched and Standard Laser-Sintered Polyamide 12 Sheets

**DOI:** 10.3390/polym13040603

**Published:** 2021-02-17

**Authors:** Anna Liebrich, Horst-Christian Langowski, Bernd R. Pinzer

**Affiliations:** 1 TUM School of Life Sciences, Technical University of Munich, 85350 Freising, Germany; h-c.langowski@tum.de; 2 Fraunhofer Institute of Process Engineering & Packaging IVV, 85354 Freising, Germany; 3 Kompetenzzentrum für Angewandte Forschung in der Lebensmittel- und Verpackungstechnologie (KLEVERTEC), 87437 Kempten, Germany; 4 Laboratory for Optical 3D Metrology and Computer Vision, University of Applied Sciences Kempten, 87435 Kempten, Germany; bernd.pinzer@hs-kempten.de

**Keywords:** powder bed fusion, laser sintering, polyamide 12, permeation properties, structural properties

## Abstract

The laser sintering of polymers is an additive manufacturing technology that is becoming increasingly established in the industrial environment. This study investigated the thickness-dependent permeation properties of laser-sintered (LS) polymers as required to design and produce components with a special barrier performance to gaseous substances. Helium and oxygen permeation experiments were carried out on quenched and standard LS polyamide 12 (PA12) sheets generated with two, four, six, and eight layers at a constant powder layer thickness of 100 µm. The structural properties of the sheets were examined by differential scanning calorimetry, light microscopy, and X-ray micro-computed tomography. A reduction in thickness resulted in higher diffusion coefficients for both types of LS sheets. An explanation could be the large volume fraction of poorly sintered powder particles adhering to the surfaces and incomplete melting and low consolidation of the polymer at small thicknesses. The thickness-dependency of the solubility coefficients was the opposite, especially for the standard LS sheets, which might be related to the larger pore volume in thicker sheets. As both effects compensated for each other, nearly constant permeation coefficients for all thicknesses were observed. The results provide further insights into different material characteristics of thin LS PA12 structures and offer new information on factors relevant to their solution and diffusion behavior.

## 1. Introduction

The importance and relevance of additive manufacturing (AM) has increased substantially over the past years. AM presently enables and facilitates flexible fabrication of complex parts and customized products in various applications [1,2,3]. Many different AM technologies exist using a wide range of different materials [4,5,6,7]. Common AM technologies for polymers are classified into the following process categories according to ISO/ASTM DIS 52900:2018: material extrusion, material jetting, binder jetting, vat photopolymerization, sheet lamination, and powder bed fusion [8]. The laser-based powder bed fusion of polymers (PBF-LB/P, according to Reference [8]) is an AM technology often termed as laser sintering of polymers. The laser sintering process consists of three main steps: first, a powder layer is deposited across the build platform and is heated to a temperature just below the melting temperature of the polymer; then, a specific cross-section of the generated parts is molten by a carbon dioxide (CO_2_) laser, while the surrounding powder acts as a supporting structure; in the third step, the build platform is lowered by the thickness of one powder layer, before the process is repeated [9].

Due to the high accuracy and the high mechanical performance of the produced parts, laser sintering is becoming increasingly established in the industrial environment [4]. However, current applications rarely include the direct manufacturing of components that are in contact with sensitive products and require a special barrier performance to different gases and vapors. This could be, for example, the fabrication of geometrically complex and individualized nozzles, grippers or casting molds for the pharmaceutical and food industries. To date, little research has been conducted regarding the performance and lifetime of laser-sintered (LS) polymers in relation to their physicochemical properties [10]. In particular, the mechanism of substance transport in thin LS polymeric structures is not well understood.

The production of thin structures in laser sintering, in general, is restricted by the spot size of the laser beam, the powder layer thickness, and the penetration depth of the laser [11,12]. Recently, several studies demonstrated that producing thin structures close to the resolution limit results in significant losses of mechanical properties. The effect equally occurred for small cross-sections in the vertical build orientation and for small numbers of consecutively sintered layers in the horizontal build orientation. The decline in material quality was explained by two different factors: first, a high volume fraction of the border zones composed of poorly sintered material and unmolten powder particles adhering to the surfaces; second, incomplete melting and low consolidation of the polymer related to detrimental sintering conditions due to the low energy densities [13,14,15,16].

Based on these findings, thin LS structures could be expected to only provide low resistance to the permeation of small gaseous molecules. Surprisingly, initial research revealed that LS polyamide 12 (PA12) sheets with a nominal thickness between 700 and 2000 µm have permeation coefficients for water vapor and oxygen in the range of those of extruded PA12 films. Despite the porous material structure, even the thinnest LS sheets investigated showed a solution–diffusion behavior as typically observed for a dense, coherent polymeric material. Independent of the build orientation, the results, however, indicated that the permeation properties of the LS sheets could depend on the thickness. The observed deviations were not significant but showed a trend that coincided well with the variation of the structural properties. It has been stipulated that the different crystallinity and crystal morphology could lead to a thickness-dependent solution and diffusion behavior [17].

This study investigates the material structure and related permeation properties of LS polymeric structures, as they form by continuously increasing the number of consecutively sintered layers. Oxygen and helium permeation experiments were carried out on LS PA12 sheets generated with two, four, six, and eight layers at a constant powder layer thickness of 100 µm. The crystallinity, porosity, and thickness distribution of the LS sheets were examined by differential scanning calorimetry (DSC), light microscopy, and X-ray micro-computed tomography (CT). In order to obtain detailed information on factors relevant to solution and diffusion processes, LS sheets were produced at two different cooling rates, i.e., rapid cooling (quenching) in contrast to slow cooling as is standard in laser sintering.

## 2. Materials and Methods

### 2.1. Production of Quenched and Standard Laser-Sintered Sheets

Disc-shaped PA12 sheets with a nominal diameter of 70 mm were produced by laser sintering of two, four, six, and eight layers at a constant powder layer thickness of 100 µm. Two different cooling rates were employed: rapid cooling (“quenched LS sheets”) and slow cooling as is standard in laser sintering (“standard LS sheets”).

Both types of LS sheets were fabricated on an EOS P110 laser sintering machine using PA2201 PA12 powder (both EOS, Electro Optical Systems GmbH, Krailling, Germany) with a mixing ratio 50/50 between virgin and recycled powder. Standard commercial system settings as provided by the laser sintering machine manufacturer were applied, which involved an alternating *x*–*y* scanning grid (hatch) with two boundary scanning lines (contour). The temperatures in the process chamber and the build chamber were set to 169 and 155 °C, respectively.

The quenched LS sheets were produced separately one after another in the center of the build platform covered with 4 mm of powder at the final build temperature. After sintering of the last layer of the parts, two further powder layers were applied, before the process was terminated. Immediately afterwards, the doors of the laser sintering machine were opened, and the LS sheets were removed, cleaned from loosely adhering powder, and placed between two thin plates of stainless steel that were previously cooled to about −78 °C in dry ice. The plates were loosely fixed with clamps in order to prevent warpage of the LSsheets. Care was taken that the applied pressure remained very low, thus excluding changes in the material structure. The LS sheets were stored between the plates for several days at 23 °C and 50% r.h. Afterwards, post-processing was performed by bead blasting in order to clean the surfaces from loosely adhering powder particles.

The standard LS sheets were randomly selected from a batch of sheets produced in stacked order with a nominal spacing of 3 mm in the build direction (*z*-direction). The overall height of applied layers was 115 mm including each 4 mm of powder deposited below and above the sintered layers to provide thermal insulation to the parts. After the building phase (~7 h), the final setup of layers was left unchanged in the build chamber and slowly cooled down to room temperature (~10 h). Then, the build envelope was removed from the laser sintering machine and the LS sheets were unpacked from the surrounding powder. Post-processing was the same as for the quenched LS sheets.

For each type of LS sheet and thickness investigated, all of the analysis steps were carried out on the same LS sheet in order to obtain precise information on possible interrelationships between different material characteristics.

### 2.2. Analysis of Structural Properties

#### 2.2.1. Differential Scanning Calorimetry

The melting transitions of the PA2201 PA12 powder and the LS sheets were determined by DSC measurements using a DSC 3+ STARe System (Mettler-Toledo GmbH, Gießen, Germany). Three individual samples with masses of 8 ± 0.5 mg were taken from each material investigated. Regarding the LS sheets, partially molten powder particles at the surfaces were carefully removed using a scalpel tool. All of the samples were heated from 23 to 230 °C at a constant rate of 10 K min^−1^ under a nitrogen atmosphere. The crystallinity was calculated by dividing the melting enthalpy of the samples by a theoretical melting enthalpy of 209.3 J g^−1^ for 100% crystalline PA12 [18].

#### 2.2.2. Light Microscopy

The crystalline structure of the LS sheets was examined using transmission light microscopy under cross-polarized conditions. This means that the sample was illuminated with linearly polarized white light and that the polarizing angle of the analyzing filter was set orthogonal to the polarization direction of the incident light. Thin microtome-cut sections with a thickness of 10 μm were prepared using a 2055 Jung Autocut rotary microtome (Leica Instruments GmbH, Nussloch, Germany). Images were taken using a Leitz Diaplan light microscope (Leitz, Wetzlar, Germany) employing a DFC 295 camera (Leica Microsystems, Wetzlar, Germany).

#### 2.2.3. X-ray Micro-Computed Tomography

**Figure 1:**
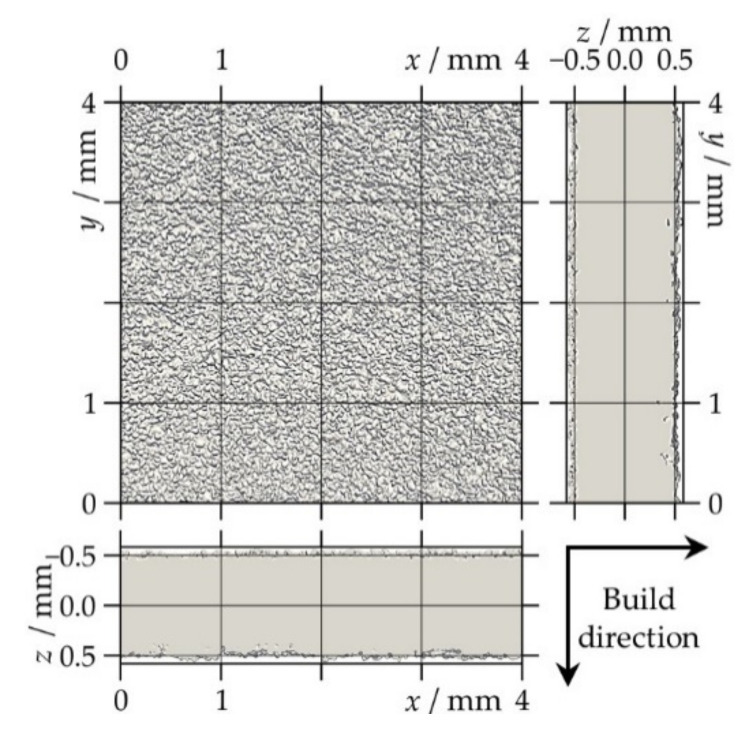
Three orthogonal views of the volume of interest for the X-ray micro-computed tomography data analysis of a laser-sintered polyamide 12 sheet generated with eight layers. The arrows indicate the build direction that corresponds to the positive *z*-direction in the two lateral views.

In order to study different porosity characteristics and the thickness distribution of the LS sheets, CT scans were carried out at Scanco headquarters (Scanco Medical AG, Brüttisellen, Switzerland) using a Scanco μCT 100 desktop device. Rectangular samples, appropriate to the size of the sample holders (cylindrical polyetherimide (PEI) tubes with 10 mm inner diameter), were manually cut out from the middle of the LS sheets. The acceleration voltage was set to 55 keV with a tube current of 109 μA, and 1500 projections were recorded per 180° sample rotation. These settings resulted in a voxel size of (4 μm)^3^. Post-processing of the raw data consisted of three steps as in Reference [19]: first, if necessary, the geometrical alignment of the scanned sample volume was slightly adjusted by rotation in three-dimensional space such that the surface was parallel to the *x*–*y* plane; next, a volume of interest (VOI) with voxel dimensions of 1000 × 1000 in the *x*- and *y*-direction times the approximate thickness of the sample in the *z*-direction was cropped (see Figure 1); finally, noise was reduced by applying a median filter (support 3), followed by Gaussian smoothing (sigma 1.2, support 3).

In order to avoid erroneous pore detection resulting from measurement noise, only pores consisting of three voxels or more (i.e., a pore volume ≥ 1.92 × 10^−7^ mm^3^) were considered in the analysis. Based on the data for pores and solid comprised in the VOI, the following metrics were calculated:

- **Porosity**: Porosity was measured as the fraction of pore volume over the total volume (=pore and solid volume).
- **Pore number density**: The pore number density was determined by dividing the number of disjoint pores by the total volume.
- **Two-dimensional (2D) porosity**: 2D porosity was defined as the fraction of pore volume over the total volume in one particular *x*–*y* slice of the VOI.
- **Effective thickness**: The effective thickness *l_eff_* as used in the permeation analysis corresponds to the average value of the thickness distribution collected from the total volume in the *z*-direction.

### 2.3. Permeation Measurements

#### 2.3.1. Sample Preparation

To avoid leakages at the sealing of samples to the permeation measurement cell, sheets of a self-adhesive aluminum foil with a circular cut-out area of 25 cm^2^ were applied on both sides of the LS sheets. In addition, a narrow strip of a two-component adhesive was applied along the fringe of the cut-out area to ensure that no gases could pass through the interface between aluminum foil and the LS sheets.

#### 2.3.2. Oxygen Permeation Measurement

The oxygen permeability was determined with an optical measurement method according to DIN 53380-5 at 23 °C and 50% r.h. using fluorescence quenching measurement equipment from PreSens (Precision Sensing GmbH, Regensburg, Germany) for time-resolved measurement of the oxygen concentration. This is a quasi-isostatic method in which both sides of the sample are maintained at atmospheric total pressure, but in which different partial pressures of the test gas oxygen prevail, with nitrogen as the second gas. To reduce the amount of oxygen dissolved in the LS sheets, they were stored in the permeation measurement cells under a nitrogen atmosphere several days prior to the measurement procedure as proposed in Reference [20].

#### 2.3.3. Helium Permeation Measurement 

The helium permeability was measured with a manometric method under a total test gas pressure difference below 100 kPa at 23 °C and 0% r.h. using a GDP-C gas permeability tester (Brugger Feinmechanik GmbH, Munich, Germany). The LS sheets were stored for several weeks in a vacuum before the permeation measurements to remove major parts of the dissolved gases.

#### 2.3.4. Evaluation of Permeation Measurements

First, the mechanism of substance transport through the LS sheets was determined from the time behavior of the permeation rate and its dependence on the pressure difference between the two sides of the sample. Gas transfer rates that are very high and, in the case of an absolute pressure difference, do not depend linearly on the pressure difference, indicate a substance transport in the form of Poiseuille or even turbulent flow [21] through pores that extend through the entire thickness of the LS sheet. The LS sheets showing this behavior were not included in the further investigations (see also Figure 10).

If this behavior could be excluded, permeation analysis was performed following the rules of a regular solution–diffusion mechanism as described by Fick’s first and second law [21,22]. It was considered that the diffusion coefficient *D* and the solubility coefficient *S* are constant values, and that according to Henry’s law, the concentrations of the permeating substance in the surface zones on the two sides of the sample depend linearly on the related external partial pressures *p_1_* and *p_2_*.

In the initial period of the permeation measurements the side of the sample through which the permeating substance emerges is effectively maintained at zero concentration, i.e., $p_{2}\approx 0$, and, therefore, $\Delta p=\left(p_{1}-p_{2}\right)\approx p_{1}=const.$ For this finite interval of time, the amount of permeated substance *Q* as a function of time *t* through a sheet of thickness *l*, which is initially at zero concentration, in one dimension is given by [22]:

(1) $$\frac{Q}{\Delta p\;l}\left[\frac{mol}{m^{2}\;Pa\;m}\right]=D\;S\left(\frac{t}{l^{2}}-\frac{1}{6\;D}-\frac{2}{\pi^{2}}\overset{\infty }{\underset{1}{\sum }}\frac{{\left(-1\right)}^{n}}{n^{2}}\exp\left(-Dn^{2}\pi^{2}t/l\right)\right)$$

Over longer periods of time, constant flow conditions are established in the stationary state until *p_2_* is no longer negligible (i.e., Δp≠const.), and the permeation rate decreases. At this stage, the rate of permeated substance per unit area *dQ*/*dt* is described by the following differential equation (see e.g., Reference [21]):

(2) $$\frac{dQ}{dt}\left[\frac{mol}{m^{2}\;s}\right]=D\;S\left(\frac{p_{1}-p_{2}}{l}\right)=P\left(\frac{p_{1}-p_{2}}{l}\right)$$

For both penetrants investigated, oxygen and helium, the evaluation of the time course of the measured permeated quantities was performed over the initial non-stationary phase and the subsequent stationary phase by applying a data fit to Equation (1) using the Scipy function “curve fit” in Python 3.7. This allowed the determination of *D* and *S* and, followed by P=D S, the calculation of the permeation coefficient *P*.

Due to the high transfer rates in the helium permeation measurements of LS sheets having small thicknesses, the time span during which Δ*p* remained virtually constant was very short. In this case, the non-stationary temporal behavior could not be resolved, and the immediate achievement of the stationary state had to be assumed. Then, only the value of the helium permeation coefficient *P_He_* could be determined by applying a data fit to Equation (2) considering a larger time interval in the stationary state.

## 3. Results and Discussion

### 3.1. Crystallinity and Crystalline Structure

**Figure 2:**
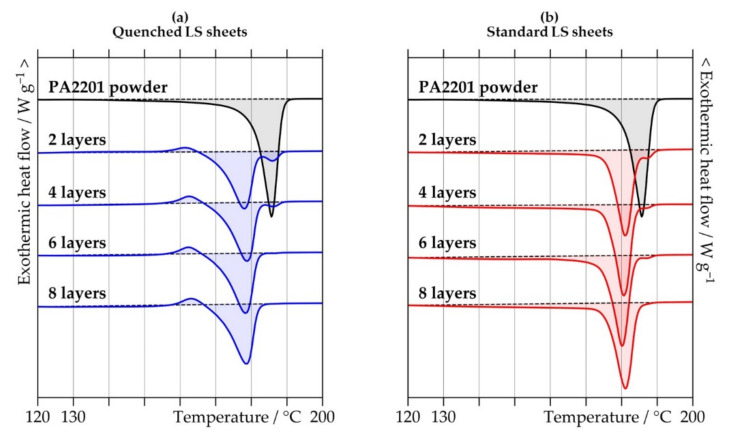
Differential scanning calorimetry curves of the first heating run of PA2201 polyamide 12 powder and (**a**) quenched and (**b**) standard laser-sintered (LS) polyamide 12 sheets generated with two, four, six, and eight layers.

Table 1 summarizes thermal properties of PA2201 PA12 powder and the two different types of LS sheets as measured by DSC. The quenched LS sheets have crystallinities ranging between 23.8% and 24.5%, and the values slightly increased with increasing thickness. The standard LS sheets show significantly higher crystallinities compared to the quenched LS sheets, with values increasing from 34.5% for two layers up to 37.0% for eight layers. At all thicknesses, the melting temperatures are slightly lower for the quenched LS sheets than for the standard LS sheets, which is in good agreement with results obtained in Reference [18], i.e., an increase in the melting temperature on annealing time of PA12 due to the improvement of crystalline order in the polymer. As can be seen in Figure 2, the quenched LS sheets all present an exothermic peak in the first heating run of the DSC measurements, suggesting recrystallization processes. This could also include the transformation among different crystal modifications that form depending on the cooling rate during crystallization of PA12 from the melt at atmospheric pressure [23,24]. For both types of LS sheets, thinner LS sheets show an additional melting peak at the melting temperature of the PA2201 PA12 powder. This bimodal melting behavior indicates the presence of powder residues that did not receive sufficient energy to completely melt, and suggests poor consolidation of the polymer when producing LS sheets with a low number of layers [25,26].

**Figure 3:**
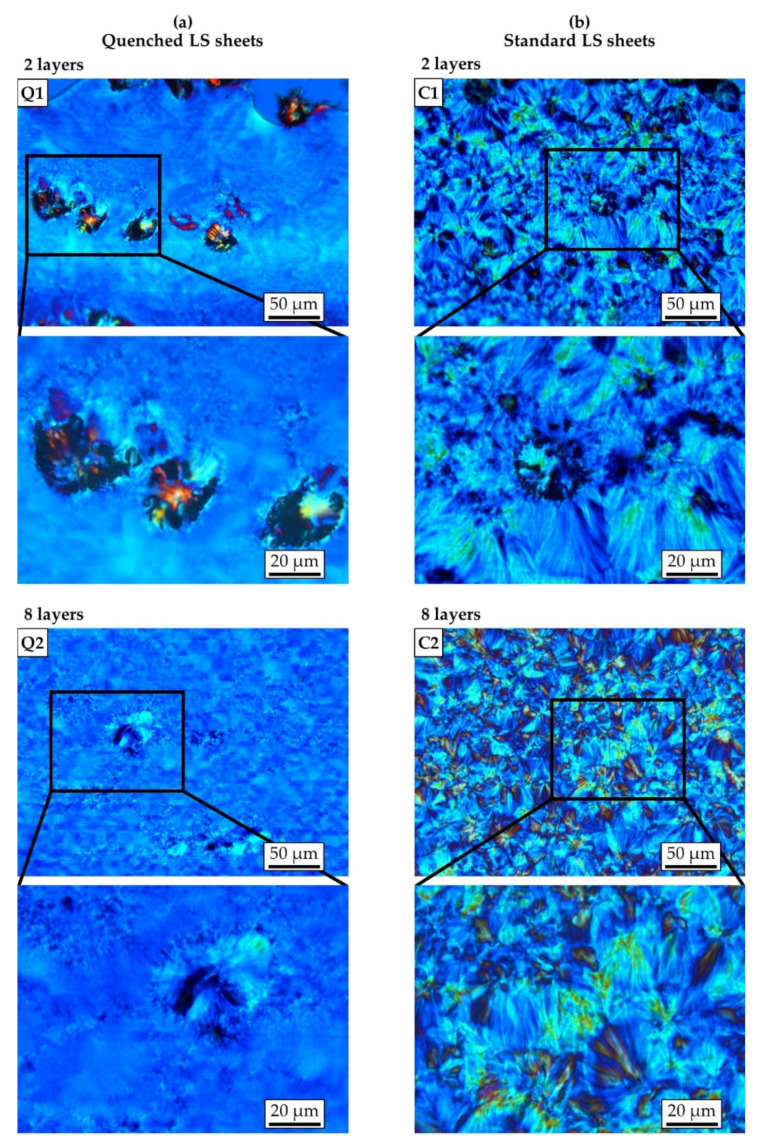
Light microscopy images of thin sections showing the inner area of (**a**) quenched and (**b**) standard laser-sintered (LS) polyamide 12 sheets generated with two and eight layers. Magnified images of the rectangular sections in the first and third row are shown in the second and fourth row.

**Figure 4:**
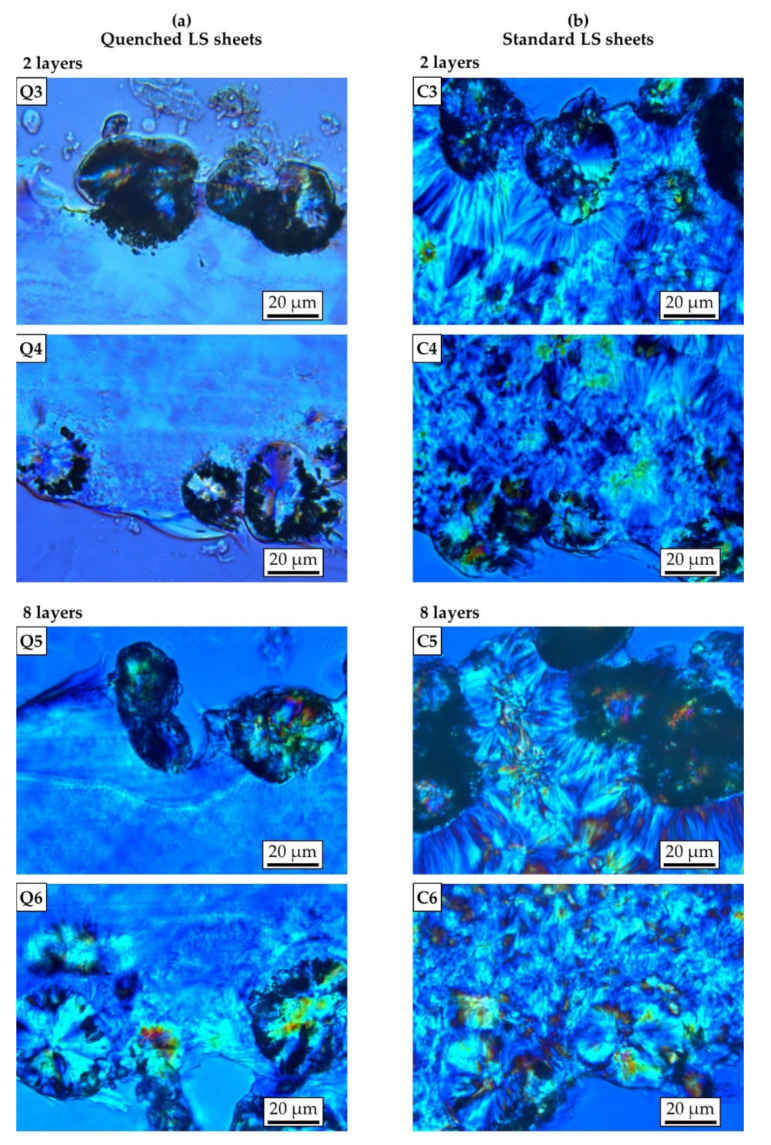
Light microscopy images of thin sections showing the top and bottom surfaces of (**a**) quenched and (**b**) standard laser-sintered (LS) polyamide 12 sheets generated with two and eight layers.

As shown by microscopic images in Figure 3 and Figure 4, the quenched LS sheets solidified in a predominantly amorphous structure, whereas the slower cooling rates of the standard LS sheets led to the formation of a distinct coarse spherulitic morphology. For both types of LS sheets, unmolten powder particles can be found at the layer interface in the LS sheets generated with two layers (Figure 3, Q1 and C1). However, while they appear in high numbers and are clearly discernable in the quenched LS sheet, they are significantly less frequent and hard to identify in the standard LS sheet. A possible explanation could be that the slower cooling of the standard LS sheets provides a longer period of time in which a temperature equilibrium between the two adjacent phases could establish. This would lead to further melting of powder particles as long as the temperature of the melt remains above the crystallization temperature of the polymer.

For the quenched LS sheets, microscopic examinations revealed an emerging crystalline structure around the adherent powder particles at the bottom surfaces, which seems to be more developed in the thicker LS sheet (Figure 4, Q4 and Q6). At the top surfaces of the quenched LS sheets, there is no visible crystallization (Figure 4, Q3 and Q5). In addition, the quenched LS sheet consisting of eight layers shows isolated crystals in the bottom area that appear at a distance similar to the powder layer thickness (Figure 3, Q2). These observations are in good agreement with previous research stating that crystallization in laser sintering would already occur even just below the powder bed surface during the building phase [27,28]. However, the notion that single crystals could have formed when the LS sheet was removed from the laser sintering machine must be considered.

Regarding the standard LS sheets, the microscopic images show that the LS sheet produced with eight layers exhibits significantly larger spherulites compared to the LS sheet consisting of two layers (Figure 3, C1 and C2). The different degree of melting of the polymer, accompanied by the influence of different temperature gradients, could have led to a different crystalline structure upon cooling [29]. Independent of the thickness, crystallization seems to be less developed in the near surface region on the bottom side of the standard LS sheets (Figure 4, C4 and C6). The effect might be explained by a low temperature in the melt and fast cooling of the polymer in the first layer. By contrast, there are large lamellae surrounding the powder particles adhering to the top surfaces of the standard LS sheets (Figure 4, C3 and C5). This is in good agreement with earlier research demonstrating that cool particles introduced into the melt during powder coating could act as crystallization nuclei [30].

### 3.2. Porosity and Pore Structure

The following section comprises an analysis of the porosity of the LS sheets referring to the closed pore structure as derived from the CT measurements. The results for overall porosity, pore number density, and different pore size characteristics are listed in Table 2.

#### 3.2.1. Overall Porosity and Pore Number Density

Both types of LS sheets show an increase in overall porosity with increasing thickness, while values range from 0.32% to 0.60% for the quenched LS sheets and from 0.31% to 1.04% for the standard LS sheets. Porosity levels are similar when comparing the two different types of LS sheets produced with two and four layers. However, the standard LS sheets show a trend of higher porosities than the quenched LS sheets for LS sheets consisting of six and eight layers.

Pore number density tends to drop with increasing thickness, while values decrease from 220 to 115 pores per mm^3^ for the quenched LS sheets and from 266 to 212 pores per mm^3^ for the standard LS sheets.

#### 3.2.2. Pore Size and Morphology

**Figure 5:**
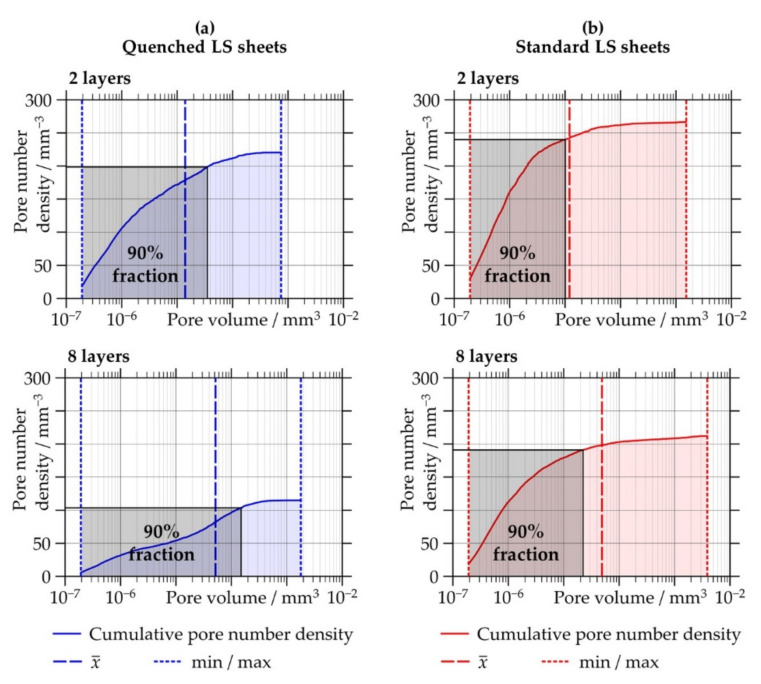
Cumulative pore number density distribution of different pore volumes of (**a**) quenched and (**b**) standard laser-sintered (LS) polyamide 12 sheets generated with two and eight layers. The black rectangles indicate the 90% fraction of the total number of detected pores.

For both types of LS sheets, the data in Table 2 reveal an increase in the average and maximum pore sizes with increasing thickness. The minimum pore sizes are the same for all of the investigated LS sheets and correspond to the detection limit of CT analysis, i.e., a pore volume of 1.92 × 10^−7^ mm^3^. The maximum pore sizes are significantly larger in the standard LS sheets than in the quenched LS sheets. The average volumes from all detected pores, however, are similar among the quenched and standard LS sheets generated with the same number of layers. An explanation can be found when looking at the number density distributions of different pore volumes presented in Figure 5. It can be observed that the major fraction of pores refers to very small pore sizes for both types of LS sheets and at all thicknesses. Apart from that, the smallest pores are even more frequent in the standard LS sheets than in the quenched LS sheets.

**Figure 6:**
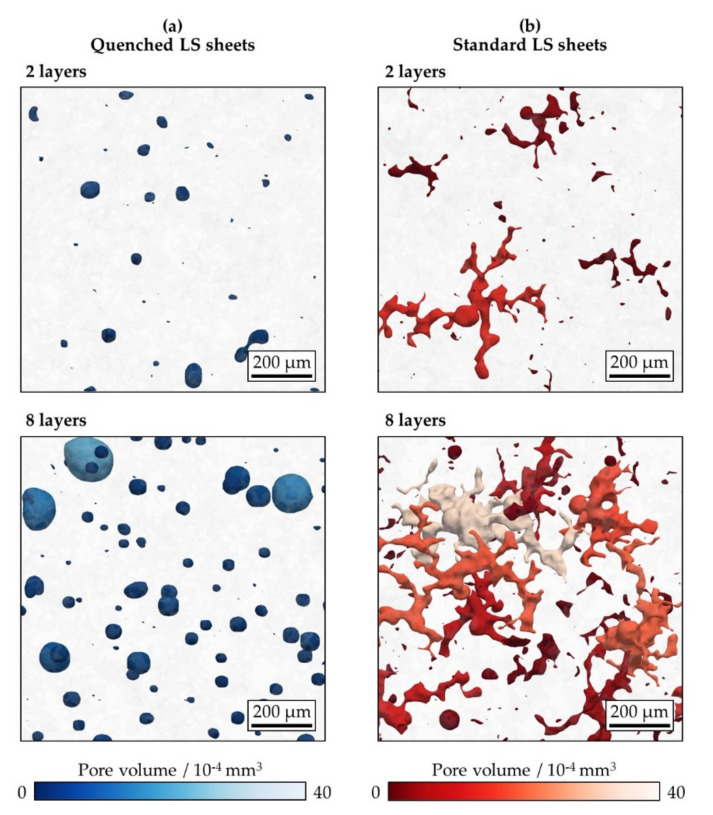
Semi-transparent view of visualized X-ray micro-computed tomography data through (**a**) quenched and (**b**) standard laser-sintered (LS) polyamide 12 sheets generated with two and eight layers. The closed pores are shown in different colors according to their volume.

As evident from the visualized CT data presented in Figure 6, pore morphology is completely different when comparing the two different types of LS sheets. The pores found in the quenched LS sheets are mainly spherical. This suggests that the polymer was in a viscous molten state when quenching was performed. In contrast, the standard LS sheets show a complex structure comprising elongated, branched pores. It was previously thought that the stretched pore shapes widely observed in LS polymers could result from the interconnection of several more compact pores that were located in close proximity [31,32]. However, the formation of pores in laser sintering and the origin of the pore morphology are not yet completely understood.

#### 3.2.3. Porosity Distribution

**Figure 7:**
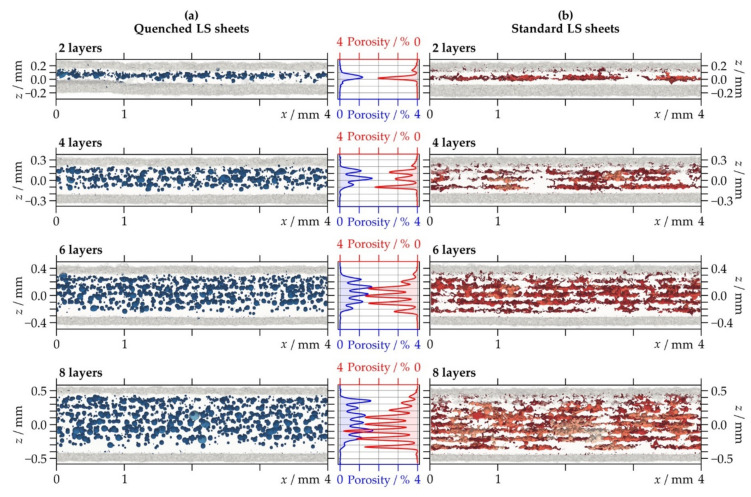
Semi-transparent view of visualized X-ray micro-computed tomography data through 4 mm (=1000 voxels) cross-sections and corresponding two-dimensional porosity distribution for (**a**) quenched and (**b**) standard laser-sintered (LS) polyamide 12 sheets generated with two, four, six, and eight layers.

Figure 7 shows the spatial distribution of pores and the calculated 2D porosity of the LS sheets. Both types of LS sheets present a repeated accumulation of pores at intervals corresponding to the powder layer thickness of 100 µm. Furthermore, the elongated pores in the standard LS sheets appear to be organized in planes parallel to the layer interfaces. The periodically coplanar porosity was observed for several different PA12 powders processed on various laser sintering machines using different parameter settings. It is thought that the migration and confluence of pores predominantly occurs within one layer or at one interface but not between successive layers [31,32,33,34,35]. When considering the dependence on thickness, the standard LS sheets show a significant increase in the porosity maxima with increasing thickness. In contrast, the quenched LS sheets present nearly equal values when comparing LS sheets produced with a different number of layers.

It is further observed from Figure 7 that all of the LS sheets possess a dense border area at both sides. This effect has been previously reported in the literature investigating porosity distribution in LS polymeric structures by means of CT [16,34,36,37]. The fusing of unmolten particles to the part surfaces [16] and the faster cooling of the border areas [36] were named as possible explanations for why less pores were detected close to the surfaces. In addition, it has been hypothesized that in the border areas only the surfaces of the particles are bonded together, while the cores remain unmolten, resulting in a material of apparently low porosity, but less integrity compared to the fully molten and recrystallized polymer [16].

### 3.3. Thickness Distribution and Effective Thickness

Table 3 shows the average value and standard deviation from the thickness distribution of the LS sheets as determined from CT data. The results were used as the effective thicknesses *l_eff_* of the LS sheets for the permeation analysis. The values, in general, are higher than those of the nominal thicknesses of the LS sheets, i.e., the number of layers times the layer thickness (=100 µm). Such an oversize typically occurs in laser sintering because the penetration depth of the laser is deeper than the powder layer thickness and can be compensated by applying rescaling factors if dimensional accuracy of parts is required [12]. When comparing the two different types of LS sheets, the average values are similar for the LS sheets produced with the same number of layers. However, with the exception of the LS sheets consisting of two layers, the standard LS sheets show slightly higher standard deviations than the quenched LS sheets, suggesting broader variation of thickness.

**Figure 8:**
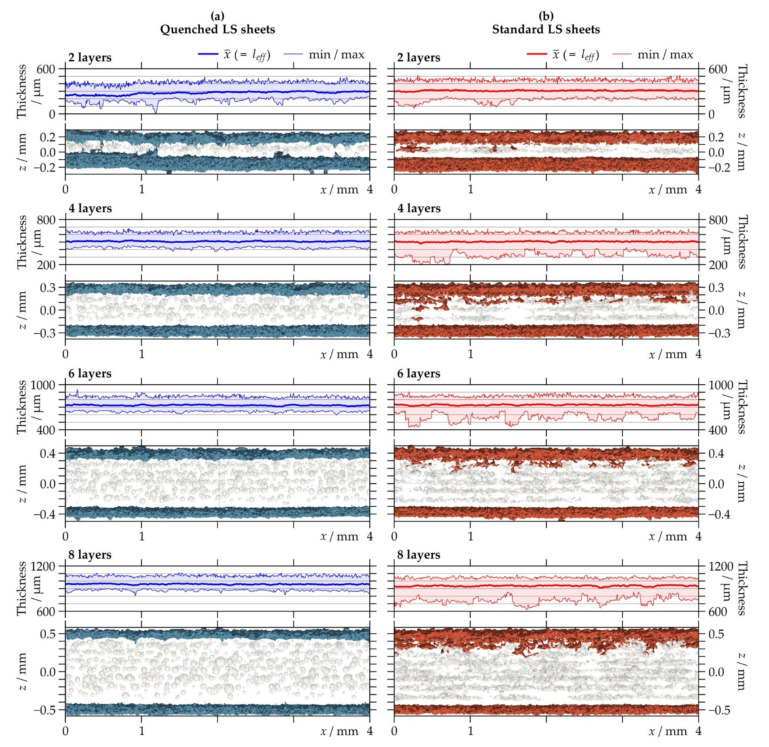
Semi-transparent view of visualized X-ray micro-computed tomography data through 4 mm (= 1000 voxels) cross-sections and projected thickness distribution with average, minimal, and maximal values of (**a**) quenched and (**b**) standard laser-sintered (LS) polyamide 12 sheets generated with two, four, six, and eight layers. The projected surface contours of the LS sheets are blue (quenched LS sheets) and red (standard LS sheets). The closed pores are shown in light gray.

**Figure 9:**
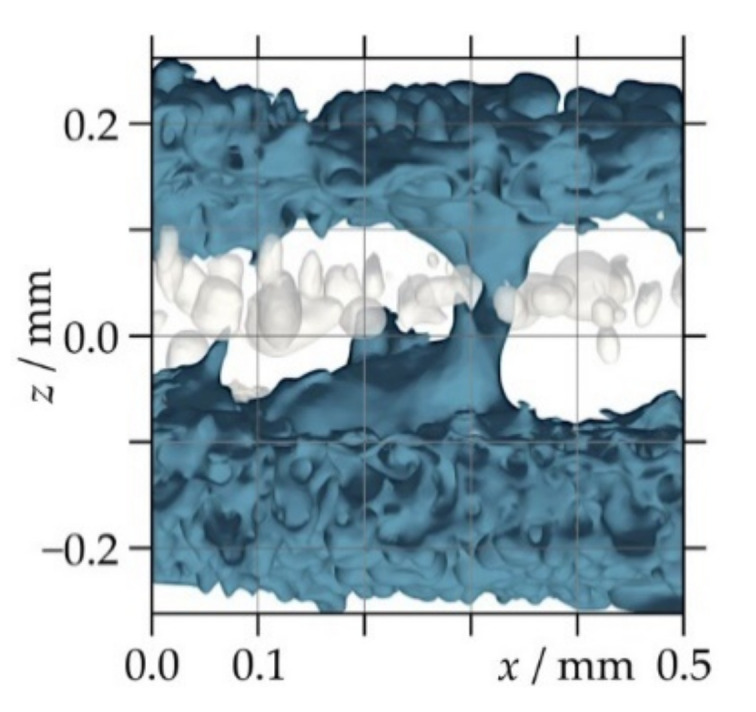
Semi-transparent view of visualized X-ray micro-computed tomography data through a 4 mm (= 1000 voxels) cross-section of a quenched laser-sintered (LS) polyamide 12 sheet generated with two layers showing a continuous pore through the LS sheet. The surface contours of the LS sheet are blue. The closed pores are shown in light gray.

Further details are given in Figure 8, which provides a 2D projection of the surface contour and the corresponding thickness profile including average, minimum, and maximum value. All of the LS sheets have an uneven surface structure, which is in line with microscopic examinations showing partially molten and unmolten powder particles adhering to the surfaces (see Section 3.1.). Furthermore, the images of the visualized CT data suggest a decrease in surface roughness on the bottom side of the LS sheets with increasing thickness. Similar findings were reported in Reference [15], where the progressive melting of adherent powder particles with increasing thickness led to a slightly smoother surface structure. Regarding the standard LS sheets, it appears that single pores close to the top edges are connected to the surfaces causing a local drop in thickness. As this is not observed for the quenched LS sheets, the effect could be related to the crystallization of the polymer. A pore extending through the entire thickness of the LS sheet (i.e., a local thickness of 0 µm), however, was only detected for the quenched LS sheet produced with a number of two layers, which is shown in enlarged representation in Figure 9.

### 3.4. Permeation Properties

**Figure 10:**
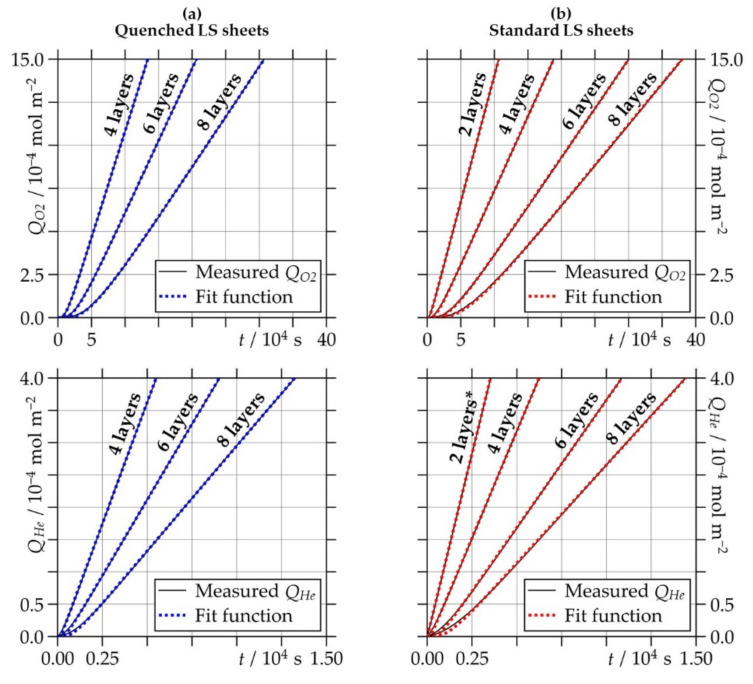
Measured permeated quantities of oxygen (*Q_O2_*) and helium (*Q_He_*) versus time (*t*) and corresponding data fits to Equation (1) or, if marked with an asterisk, to Equation (2) for (**a**) quenched and (**b**) standard laser-sintered (LS) polyamide 12 sheets generated with two, four, six, and eight layers.

**Figure 11:**
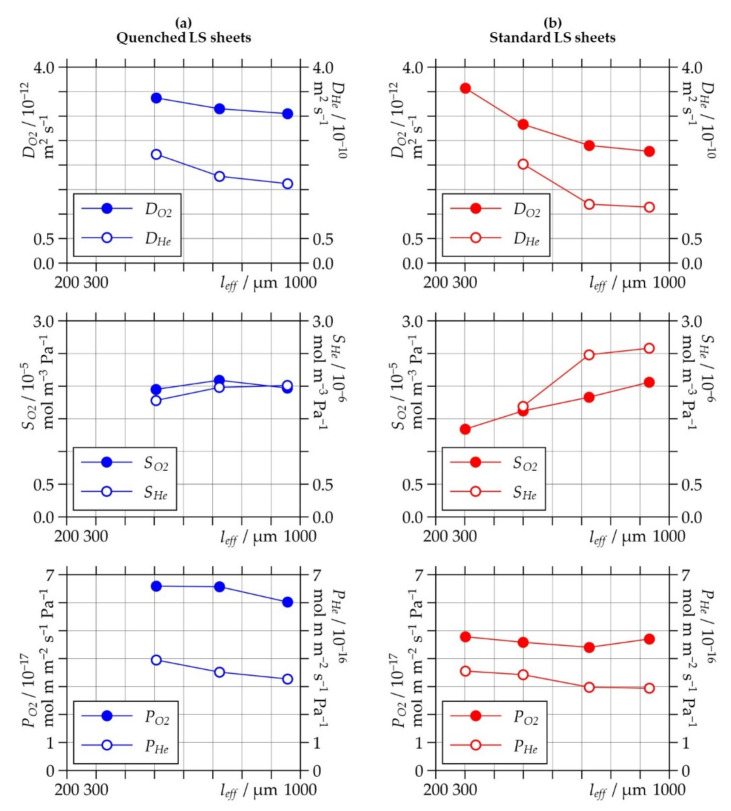
Oxygen and helium diffusion coefficients (*D_O2_*/*D_He_*), solubility coefficients (*S_O2_*/*S_He_*), and permeation coefficients (*P_O2_*/*P_He_*) as a function of the effective thickness (*l_eff_*) of (**a**) quenched and (**b**) standard laser-sintered (LS) polyamide 12 sheets generated with two, four, six, and eight layers.

As already expected from the CT results, the quenched LS sheet consisting of only two layers shows permeation behavior, indicating a real flow of substances for both penetrants, oxygen and helium. For the remaining LS sheets, however, no measurable flow through continuous pores is observed. They all show permeation behavior that could be well described by the theory of solution–diffusion as typically for the transport of low molecular weight substances in dense and coherent polymeric sheets (see Section 2.3.4.). This is demonstrated in Figure 10, which shows the time course of the measured permeated quantities of oxygen *Q_O2_* and helium *Q_He_* and the corresponding data fits to Equation (1) for all of the LS sheets included in the permeation analysis. Regarding the standard LS sheet produced with two layers, the helium permeation experiment did not yield enough data to adequately describe the non-stationary flow regime, and only *P_He_* could be deduced from the measurement curves using a data fit to Equation (2). The results, where possible for *D* and *S*, and for *P* are summarized in Table 4 and shown as a function of *l_eff_* of the LS sheets in Figure 11.

Both types of LS sheets show a similar increase in the diffusion coefficients *D_O2_* and *D_He_* with decreasing thickness of the LS sheets. The values, in general, are higher for the quenched LS sheets than for the standard LS sheets, which corresponds to the well-known fact that a higher degree of crystallinity reduces diffusive transport of substances in semi-crystalline polymers [38,39]. Regarding the standard LS sheets, the thickness-dependent diffusion rates could be related to the different crystallinity and crystalline structure when comparing LS sheets produced with different numbers of layers. However, this explanation is less valid for the quenched LS sheets, which all show a predominantly amorphous structure with only small variation of crystallinity. Based on the results, the effect might be due to the factors also responsible for the reduction in mechanical performance of LS polymers at small thicknesses: the larger volume fraction of the poorly sintered border zones compared to thicker LS sheets and moreover, low material integrity due to incomplete melting and low consolidation of the polymer (see also Section 1).

Regarding the results for the solubility coefficients *S_O2_* and *S_He_*, the quenched LS sheets show values for both gases that are virtually independent of the thickness. In contrast, the values significantly increase with increasing thickness for the standard LS sheets. At higher numbers of layers, the solubility coefficients of the standard LS sheets are even above those of the quenched LS sheets. This is somewhat surprising, since crystallization, in general, tends to reduce solubility of simple gases in polymers [38,39]. These findings strongly suggest that the solubility behavior of LS polymers could depend on the pore volume. Moreover, the results for *S_He_* could lead to the hypothesis that small nanometer-sized pores, which are not detectable by light microscopy or CT, are also present in significant amounts. The observed progressive crystallization of the polymer at higher thicknesses could lead to the formation of such additional microcavities that may absorb more gaseous helium.

As the thickness-dependencies of the diffusion coefficients and the solubility coefficients are opposite, especially in the standard LS sheets, both effects compensate for each other and finally lead to permeation coefficients that are nearly independent of the thickness.

## 4. Conclusions

This study investigated the thickness-dependent permeation behavior of LS polymeric structures as shown by thin PA12 sheets produced at two different cooling rates, i.e., rapid cooling (“quenched LS sheets”) and slow cooling as is standard in laser sintering (“standard LS sheets”).

Regarding the structural properties of the fabricated LS sheets, they all presented a material structure according to the pressure-less and layer-wise manufacturing, which is a dense border of poorly sintered and unmolten powder particles surrounding an inner porous structure where pores appear at intervals according to the powder layer thickness. Apart from that, the different cooling rates led to substantially different crystallinity and porosity characteristics: the quenched LS sheets showed a predominantly amorphous structure and nearly spherical pores, whereas the standard LS sheets presented a course spherulitic morphology with branched pores aligned coplanar to the layer interfaces. When comparing LS sheets generated with different numbers of layers, both types of LS sheets showed an increase in crystallinity and porosity with increasing thickness. Nevertheless, the effect was more pronounced for the standard LS sheets than for the quenched LS sheets. This indicates that besides the layer-wise manufacturing, the slow cooling rate in standard laser sintering could promote a thickness-dependent material structure.

For both types of LS sheets, the permeation analysis disclosed different diffusion and solubility coefficients for oxygen and helium depending on the thickness. Due to the different structural properties, the transport of gaseous substances greatly differed when comparing the quenched and the standard LS sheets. By relating the permeation results to the structural properties, it was possible to identify factors relevant to diffusion and solution processes and, based on this, to gain a better understanding of the thickness-dependent permeation behavior of LS polymeric structures. The main findings and related conclusions can be summarized as follows:

- Quenched and standard LS sheets showed a similar increase in diffusion coefficients with decreasing thickness. The main source for this behavior thus might be the high volume fraction of the border zones constituted by poorly sintered powder particles as well as incomplete melting and poor consolidation of the polymer and therefore low material integrity when producing very thin structures.
- The standard LS sheets showed a significant increase in solubility coefficients with increasing thickness. This suggested that the amount of absorbed gases could depend on the pore volume including additional nanometer-sized pores that are not detectable by light microscopy or CT.
- Opposite behavior of the diffusion and solubility coefficients, especially for the standard LS sheets, led to virtually unchanged values of the permeation coefficients for all thicknesses.

## Figures and Tables

**Table 1:** Thermal properties of PA2201 polyamide 12 powder and quenched and standard laser-sintered (LS) polyamide 12 sheets generated with two, four, six, and eight layers. Values are the average from three individual samples with the standard deviation shown in brackets.

Number of Layers		2	4	6	8
Crystallinity/%	PA2201 powder	-------------------------------- 50.8 (4.6) --------------------------------			
	Quenched LS sheets	23.8 (1.7)	24.0 (1.0)	24.4 (0.2)	24.5 (1.2)
	Standard LS sheets	34.5 (0.7)	35.2 (2.5)	37.0 (0.6)	37.0 (0.7)
Recrystallization temperature/°C	PA2201 powder	----------------------------- Not observed -----------------------------			
	Quenched LS sheets	161.7 (0.6)	162.4 (0.0)	162.2 (0.0)	163.5 (1.0)
	Standard LS sheets	Not observed	Not observed	Not observed	Not observed
Melting temperature/°C	PA2201 powder	------------------------------ 185.5 (0.6) -------------------------------			
	Quenched LS sheets	178.7 (2.0)	178.9 (0.2)	178.8 (0.9)	178.7 (0.4)
	Standard LS sheets	180.8 (0.4)	180.7 (0.1)	180.0 (0.5)	181.1 (0.9)

**Table 2:** Porosity, pore number density, and average value ($\bar{x}$), standard deviation (*s*), and maximum value (max) of pore volumes measured by X-ray micro-computed tomography (CT) for quenched and standard laser-sintered (LS) polyamide 12 sheets generated with two, four, six, and eight layers.

Number of Layers			2	4	6	8
Porosity/%		Quenched LS sheets	0.32	0.45	0.56	0.60
		Standard LS sheets	0.31	0.47	0.78	1.04
Pore number density/mm^−3^		Quenched LS sheets	220	144	152	115
		Standard LS sheets	266	231	182	212
Pore volume/10^−4^ mm^3^	$\bar{x}$	Quenched LS sheets	0.14	0.31	0.37	0.52
		Standard LS sheets	0.12	0.20	0.43	0.49
	*s*	Quenched LS sheets	0.43	0.69	0.71	1.01
		Standard LS sheets	0.83	1.26	1.80	2.84
	max *	Quenched LS sheets	7.48	8.51	6.09	17.96
		Standard LS sheets	15.29	23.14	20.43	38.87

* Minimum values for all of the investigated LS sheets correspond to the detection limit of the CT analysis, which is a pore volume of 1.92 × 10^−7^ mm^3^.

**Table 3:** Average value ($\bar{x}$ = *l_eff_*) and standard deviation (*s*) of the thickness distribution of quenched and standard laser-sintered (LS) polyamide 12 sheets generated with two, four, six, and eight layers.

Number of Layers			2	4	6	8
Thickness/µm	$\bar{x}$ (=*l_eff_*)	Quenched LS sheets	269	506	723	956
		Standard LS sheets	301	500	726	932
	*s*	Quenched LS sheets	44	37	37	36
		Standard LS sheets	41	46	43	49

**Table 4:** Oxygen and helium diffusion coefficients (*D_O2_*/*D_He_*), solubility coefficients (*S_O2_*/*S_He_*), and permeation coefficients (*P_O2_*/*P_He_*) of quenched and standard laser-sintered (LS) polyamide 12 sheets generated with two, four, six, and eight layers.

Number of Layers		2	4	6	8
*D_O2_*/10^−12^ m^2^ s^−1^	Quenched LS sheets	-	3.4	3.1	3.0
	Standard LS sheets	3.6	2.8	2.4	2.3
*D_He_*/10^−10^ m^2^ s^−1^	Quenched LS sheets	-	2.2	1.8	1.6
	Standard LS sheets	-	2.0	1.2	1.1
*S_O2_*/10^−5^ mol m^−3^ Pa^−1^	Quenched LS sheets	-	2.0	2.1	2.0
	Standard LS sheets	1.3	1.6	1.8	2.1
*S_He_*/10^−6^ mol m^−3^ Pa^−1^	Quenched LS sheets	-	1.8	2.0	2.0
	Standard LS sheets	-	1.7	2.5	2.6
*P_O2_*/10^−17^ mol m m^−2^ s^−1^ Pa^−1^	Quenched LS sheets	-	6.6	6.6	6.0
	Standard LS sheets	4.8	4.6	4.4	4.7
*P_He_*/10^−16^ mol m m^−2^ s^−1^ Pa^−1^	Quenched LS sheets	-	3.9	3.5	3.3
	Standard LS sheets	3.6	3.4	3.0	2.9

## References

[1] Berman B. (2012). 3-D printing: The new industrial revolution. Bus. Horiz..

[2] Attaran M. (2017). The rise of 3-D printing: The advantages of additive manufacturing over traditional manufacturing. Bus. Horiz..

[3] Tofail S.A., Koumoulos E.P., Bandyopadhyay A., Bose S., O’Donoghue L., Charitidis C. (2018). Additive manufacturing: Scientific and technological challenges, market uptake and opportunities. Mater. Today.

[4] Gebhardt A., Kessler J., Thurn L. (2018). 3D Printing: Understanding Additive Manufacturing.

[5] Bai Y., Wagner G., Williams C.B. (2017). Effect of Particle Size Distribution on Powder Packing and Sintering in Binder Jetting Additive Manufacturing of Metals. J. Manuf. Sci. Eng..

[6] Hwang H.H., Zhu W., Victorine G., Lawrence N., Chen S. (2018). 3D-Printing of Functional Biomedical Microdevices via Light-and Extrusion-Based Approaches. Small Methods.

[7] Zeng M., Zhang Y. (2019). Colloidal nanoparticle inks for printing functional devices: Emerging trends and future prospects. J. Mater. Chem. A.

[8] (2018). ISO/ASTM DIS 52900:2018, Additive Manufacturing—General Principles—Terminology.

[9] Goodridge R., Tuck C., Hague R. (2012). Laser sintering of polyamides and other polymers. Prog. Mater. Sci..

[10] Schmid M. (2018). Laser Sintering with Plastics: Technology, Processes, and Materials.

[11] Wegner A., Witt G. (2012). Design rules for laser sintering. J. Plast. Technol..

[12] Adam G.A.O., Zimmer D. (2015). On design for additive manufacturing: Evaluating geometrical limitations. Rapid Prototyp. J..

[13] Tasch D., Mad A., Stadlbauer R., Schagerl M. (2018). Thickness dependency of mechanical properties of laser-sintered polyamide lightweight structures. Addit. Manuf..

[14] Wörz A., Drummer D. Understanding hatch-dependent part properties in SLS. Proceedings of the 29th Annual International Solid Freeform Fabrication Symposium—An Additive Manufacturing Conference.

[15] Wörz A., Wudy K., Drummer D. Understanding the influence of energy-density on the layer dependent part properties in laser-sintering of PA12. Proceedings of the 30th Annual International Solid Freeform Fabrication Symposium—An Additive Manufacturing Conference.

[16] Sindinger S.-L., Kralovec C., Tasch D., Schagerl M. (2020). Thickness dependent anisotropy of mechanical properties and inhomogeneous porosity characteristics in laser-sintered polyamide 12 specimens. Addit. Manuf..

[17] Liebrich A., Langowski H.-C., Pinzer B.R. Effect of thickness and build orientation on the water vapor and oxygen permeation properties of laser-sintered polyamide 12 sheets. Rapid Prototyp. J..

[18] Gogolewski S., Czerntawska K., Gastorek M. (1980). Effect of annealing on thermal properties and crystalline structure of polyamides. Nylon 12 (polylaurolactam). Colloid Polym. Sci..

[19] Liebrich A., Langowski H., Schreiber R., Pinzer B. (2019). Porosity distribution in laser-sintered polymeric thin sheets as revealed by X-ray micro tomography. Polym. Test..

[20] Müller K., Scheuerer Z., Florian V., Skutschik T., Sängerlaub S. (2017). Comparison of test methods for oxygen permeability: Optical method versus carrier gas method. Polym. Test..

[21] Barrer R.M. (1941). Diffusion in and through Solids.

[22] Coulson C.A., Crank J. (1958). The Mathematics of Diffusion. Math. Gaz..

[23] Hiramatsu N., Haraguchi K., Hirakawa S. (1983). Study of Transformations among α, γ and γ’Forms in Nylon 12 by X-Ray and DSC. Jpn. J. Appl. Phys..

[24] Fischer C., Seefried A., Drummer D. (2016). Crystallization and Component Properties of Polyamide 12 at Processing-Relevant Cooling Conditions. Polym. Eng. Sci..

[25] Zarringhalam H., Majewski C., Hopkinson N. (2009). Degree of particle melt in Nylon-12 selective laser-sintered parts. Rapid Prototyp. J..

[26] Kruth J.-P., Levy G., Klocke F., Childs T. (2007). Consolidation phenomena in laser and powder-bed based layered manufacturing. CIRP Ann..

[27] Zhao M., Wudy K., Drummer D. (2018). Crystallization Kinetics of Polyamide 12 during Selective Laser Sintering. Polymers.

[28] Amado A., Wegener K., Schmid M., Levy G. Characterization and modeling of non-isothermal crystallization of Polyamide 12 and co-Polypropylene during the SLS process. Proceedings of the 5th International Polymers & Moulds Innovations Conference.

[29] Balemans C., Looijmans S.F., Grosso G., Hulsen M.A., Anderson P.D. (2020). Numerical analysis of the crystallization kinetics in SLS. Addit. Manuf..

[30] Drummer D., Greiner S., Zhao M., Wudy K. (2019). A novel approach for understanding laser sintering of polymers. Addit. Manuf..

[31] Stichel T., Frick T., Laumer T., Tenner F., Hausotte T., Merklein M., Schmidt M. (2017). A Round Robin study for Selective Laser Sintering of polyamide 12: Microstructural origin of the mechanical properties. Opt. Laser Technol..

[32] Stichel T., Frick T., Laumer T., Tenner F., Hausotte T., Merklein M., Schmidt M. (2018). A Round Robin study for selective laser sintering of polymers: Back tracing of the pore morphology to the process parameters. J. Mater. Process. Technol..

[33] Dupin S., Lame O., Barrès C., Charmeau J.-Y. (2012). Microstructural origin of physical and mechanical properties of polyamide 12 processed by laser sintering. Eur. Polym. J..

[34] Dewulf W., Pavan M., Craeghs T., Kruth J.-P. (2016). Using X-ray computed tomography to improve the porosity level of polyamide-12 laser sintered parts. CIRP Ann..

[35] Bain E.D., Garboczi E.J., Seppala J.E., Parker T.C., Migler K.B. (2019). AMB2018-04: Benchmark Physical Property Measurements for Powder Bed Fusion Additive Manufacturing of Polyamide 12. Integr. Mater. Manuf. Innov..

[36] Rüsenberg S., Schmidt L., Schmid H. Mechanical and Physical Properties–A Way to assess quality of Laser Sintered Parts. Proceedings of the 22nd International Solid Freeform Fabrication Symposium—An Additive Manufacturing Conference.

[37] Pavan M., Craeghs T., Verhelst R., Ducatteeuw O., Kruth J.-P., Dewulf W. (2016). CT-based quality control of Laser Sintering of Polymers. Case Stud. Nondestruct. Test. Eval..

[38] Rogers C., Comyn J. (1985). Permeation of Gases and Vapours in Polymers. Polymer Permeability.

[39] Van Krevelen D.W., Te Nijenhuis K. (2009). Properties of Polymers: Their Correlation with Chemical Structure; Their Numerical Estimation and Prediction from Additive Group Contributions.

